# Extensive central nervous system involvement in Merkel cell carcinoma: a case report and review of the literature

**DOI:** 10.1186/1752-1947-5-35

**Published:** 2011-01-26

**Authors:** Kasim Abul-Kasim, Kristina Söderström, Lennart Hallsten

**Affiliations:** 1 Faculty of Medicine, Lund University, Diagnostic Centre for Imaging and Functional Medicine, Skåne University Hospital, 205 02 Malmö, Sweden; 2 Department of Oncology, Skåne University Hospital, 205 02 Malmö, Sweden

## Abstract

**Introduction:**

Merkel cell carcinoma is a rare malignant cutaneous neoplasm that is locally invasive and frequently metastasizes to lymph nodes, liver, lungs, bone and brain. The incidence of Merkel cell carcinoma has increased in the past three decades.

**Case presentation:**

A 65-year-old Caucasian man presented with a sudden onset of severe headache and a three-month history of balance disturbance. Magnetic resonance imaging revealed a large meningeal metastasis. The radiologic workup showed retroperitoneal and inguinal lymph node metastases. Biopsy of the inguinal lymph nodes showed metastases of Merkel cell carcinoma. Biopsy from three different suspected skin lesions revealed no Merkel cell carcinoma, and the primary site of Merkel cell carcinoma remained unknown. Leptomeningeal metastases, new axillary lymph node metastases, and intraspinal (epidural and intradural) metastases were detected within six, seven and eight months, respectively, from the start of symptoms despite treating the intracranial metastasis with gamma knife and the abdominal metastases with surgical dissection and external radiotherapy. This indicates the aggressive nature of the disease.

**Conclusion:**

To the best of our knowledge, this is the first report in the literature of an intracranial meningeal metastasis of Merkel cell carcinoma treated with gamma knife and of intraspinal intradural metastases of Merkel cell carcinoma. Despite good initial response to radiotherapy, recurrence and occurrence of new metastases are common in Merkel cell carcinoma.

## Introduction

Merkel cell carcinoma (MCC) is a rare malignant neoplasm of the skin that is locally invasive and frequently metastasizes to lymph nodes, liver, lungs, bone and brain [1]. The tumor was first described by Toker in 1972 [2] as a trabecular cell carcinoma. The fact that MCC is now considered a neuroendocrine tumor is supported by the presence of calcitonin and neuron-specific enolase within the tumor [1]. The diagnosis of MCC is based on the clinical findings of aggressive cutaneous tumors and the histopathologic examination of specimens using light and electron microscopy with a defined panel of immunoperoxidase stains [3]. Hodgson [4] reported that the incidence of MCC has increased threefold between 1986 and 2001 (the rate of MCC increased from 0.15 cases per 100,000 in 1986 to 0.44 cases per 100,000 in 2001) [4]. MCC often affects elderly patients with a mean age at presentation of about 75 years [5]. The head and neck are the most common sites affected by MCC followed by the legs [6]. A review of the literature showed a 27% to 60% incidence of local recurrence, a 45% to 91% incidence of lymph node metastases and an 18% to 52% incidence of distant metastases [3].

We report a case of MCC with extensive central nervous system (CNS) metastases with (1) intracranial meningeal, (2) intraspinal epidural and (3) intraspinal intradural metastases. A literature review of the reported cases of the intracranial and the intraspinal spread of MCC was also performed and presented.

## Case presentation

**Figure 1:**
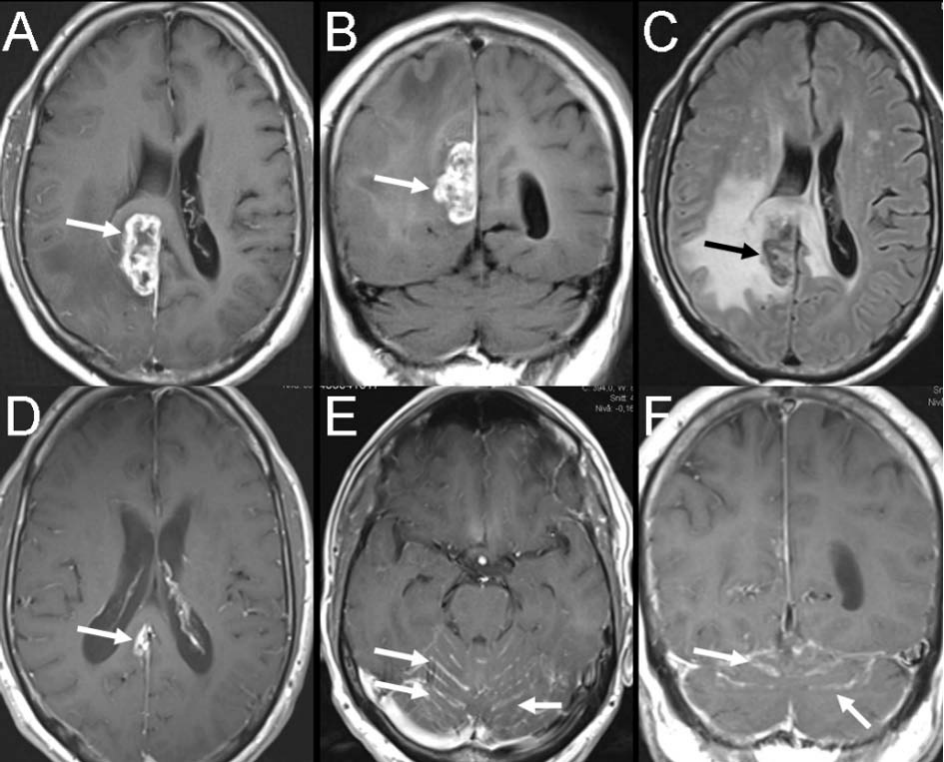
**Axial magnetic resonance imaging (MRI) scan at three different occasions**. Images (**A-C**) show the initial MRI with a large right-sided supratentorial meningeal tumor (arrows) with extensive surrounding edema (bright signal, **C**). **D) **MRI after treatment with gamma knife shows marked reduction of the tumor size with only little residual tumor (arrow). **E-F) **MRI six months from the start of symptoms shows leptomeningeal metastases with linear contrast enhancement along the cerebellar sulci (arrows).

A 65-year-old Caucasian man presented with a sudden onset of severe headache and with a three-month history of balance disturbance. Apart from a pathologic finger-nose test result and positive Grasset test result on the left side, no other physical or neurologic abnormalities were found. Computed tomography (CT) showed a large 4 cm, right-sided parietal parasagittal tumor. Magnetic resonance imaging (MRI) showed a large 4 cm, inhomogeneously enhancing mass that exhibited a wide falx attachment and dural tail. The meningeal mass was surrounded by extensive edema of the right parietal lobe (Figure 1A-C). Cerebral blood volume (CBV) map showed high intratumoral CBV. MR spectroscopy showed high choline:*N*-acetylaspartate and choline:creatine ratios (9.5 and 15, respectively) as well as occurrence of lactate and lipid peak in the tumor but not in the surrounding edema. Although the morphologic findings of the supratentorial mass on conventional MRI were consistent with meningioma, the occurrence of the extensive edema and the MR spectroscopy findings were highly suggestive of metastasis, and our final radiologic diagnosis was meningeal metastasis. The patient was admitted to the neurology department, and immediate treatment with cortisone therapy was started. A search for the primary tumor with CT and FDG-PET (fluorodeoxyglucose positron emission tomography) revealed lymph node enlargement and increased FDG uptake in the left inguinal region, iliac, aortocaval and paraaortic regions. Needle biopsy was obtained from the enlarged lymph nodes in the left inguinal region. Removal of three skin lesions in the left lower limb showed no definite primary tumor.

The large supratentorial meningeal tumor was treated with gamma knife. MRI control 13 days after treatment with gamma knife showed marked reduction of the volume of the intracranial meningeal tumor (Figure 1D). Retroperitoneal and inguinal lymph node dissection was performed. Histopathology showed small cell carcinoma consistent with MCC (immunohistopathologic analysis was positive for the epithelial markers [AE1/AE3 and CK20] and neurofilament but negative for lymphoma and melanoma markers [CD45 and HMB45, respectively]). Cerebrospinal fluid cytology also showed MCC.

**Figure 2:**
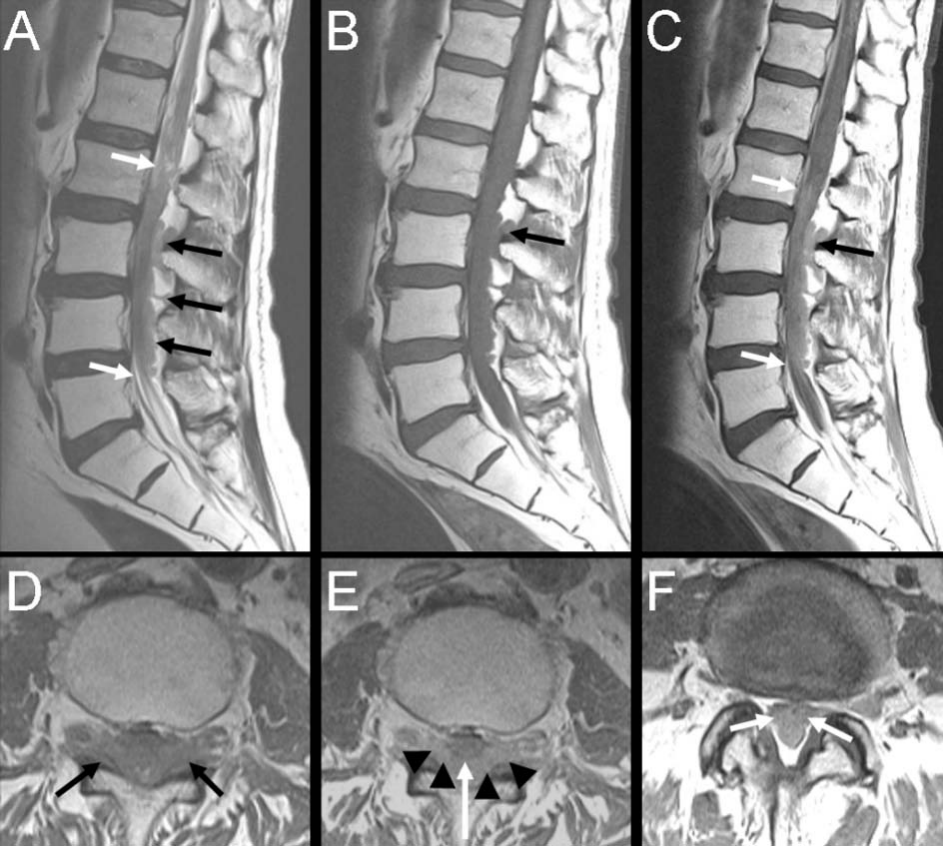
**Magnetic resonance imaging (MRI) scan of the lumbar spine eight months after the onset of symptoms**. T2-weighted (**A**) and T1-weighted (**B-C**) images before and after contrast administration show mild contrast enhancing multilobular tumors in the epidural fat behind the dural sac at the level of L3-L4 (black arrows) and a dural sac filled with intradural tumors (white arrows). Note the absence of the normal cerebrospinal fluid signal in the dural sac below the medullary conus. Axial T1-weighted images before (**D**) and after (**E**) contrast administration show the epidural metastases lateral and dorsal to the dural sac (black arrows in D; arrowheads in E). The white arrow in E shows the dorsal limit of the dural sac. **F) **Axial T1-weighted image after contrast administration shows a tumor-filled dural sac (white arrows).

About three months after the retroperitoneal and inguinal lymph node dissection, the patient received 40 Gy of external radiation for the paraaortal and iliac retroperitoneal lymph node metastasis and 50.9 Gy for the metastasis in the left inguinal region. Thereafter, FDG-PET showed total regression of the FDG uptake in the lymph nodes, which previously had shown increased uptake. A planned MRI of the brain about six months after the onset of symptoms showed evidence of cerebellar leptomeningeal enhancement (Figures 1E and 1F), which was immediately treated with 30 Gy of palliative radiation therapy. FDG-PET study approximately seven months after the onset of symptoms showed a new enlarged left-sided axillary lymph node with increased FDG uptake. Approximately eight months after the onset of symptoms, the patient was admitted for increasing back pain and a four-day history of rapidly progressing weakness of the lower limbs that required the patient to start using a wheelchair. On examination, lower limb weakness, hyporeflexia and a positive Babinski sign were found. Emergency MRI of the spine and the spinal canal showed that the dural sac between the first and fifth lumbar vertebra was filled with intradural tumor masses with mild contrast enhancement (Figures 2A to 2F). There were multiple tumor masses in the epidural space with extension into several lumbar and lower thoracic neural foramens. Because the patient's general condition deteriorated rapidly, further radiation therapy against the intraspinal tumors was not possible. The patient died two weeks after the detection of the intraspinal tumors. At autopsy, lung metastases were found, but there was no evidence of residual macroscopic intracranial tumor and no metastases to the vertebral column. Unfortunately, some technical difficulties restrained the examination of the spinal canal.

## Discussion

This case report initially showed a large supratentorial meningeal metastasis and later showed cerebellar leptomeningeal metastases of MCC. In addition, inguinal and retroperitoneal metastases were found, but the site of the patient's primary MCC remained unknown. Hitherto, only 18 cases of intracranial metastases of MCC have been reported, of which 15 cases were reviewed by Feletti *et al. *[7]. Table 1 shows the heterogeneity of treatment of MCC brain metastases because there is no evidence-based first choice of treatment described in the literature for this type of brain metastasis. We believe that until a considerable amount of case reports are available in the literature, the choice of treatment of MCC brain metastases will depend, as in many other types of brain metastases, on the number of metastases, systemic spread of the disease and the accessibility to surgery. We believe that the CNS metastases of MCC are an expression of a hematogenous systemic spread of the disease. Barkdull *et al. *[8] postulated that an intracranial spread from MCC of the frontal scalp was through communicating veins rather than local destructive process. Perineural spread from the head and neck tumor has also been suggested [9].

**Table 1:** Compiled from the literature review recently presented by Feletti et al [7] and a few other reports

Total number of patients reported	18, including our case report
**Age, range**	45-71 years

**Gender: male/female**	12/4 (two not defined)

**Location of CNS metastases**	
Parietal lobe	3
Meningeal	3
Cerebellum	2
Frontal	1
Frontoparietal	1
Hypophysis and cavernous sinus	1
Brainstem	1
Not defined	6

**Other metastasis**	13 of 18
Lymph nodes	8
Lungs	2
Liver	1
Choroidal	1
Cutaneous	1

**Time since the detection of the primary tumor**	0-4 years (50% <1 year)

**Treatment for brain metastasis**	
Radiotherapy + chemotherapy	5
Surgical removal + radiotherapy + chemotherapy	4
Radiotherapy only	2
Surgical removal only	1
Chemotherapy only	1
Gamma knife	1
Not defined	4

**Survival after diagnosis**	1 month to >3 years

This is the first report in the literature of intracranial metastasis of MCC that was treated with gamma knife, although the primary indication at the time of gamma knife surgery was removal of a large meningeal tumor of unknown origin. The patient and his relatives arranged the gamma knife treatment at another institution because the neurosurgeons in our regional institution regarded the tumor as meningioma with no need for rapid surgical intervention even though the radiologic report raised the suspicion of meningeal metastases. Based on the knowledge of the occurrence of lymph node metastases, the treating oncologists and surgeons aimed to treat the meningeal tumor with a less invasive method (treatment with gamma knife surgery instead of conventional surgery). In the past few years, the role of gamma knife surgery in the treatment of brain metastasis has proven to be associated with a longer survival time and better local tumor control in lung cancer metastases [10] and melanoma metastases [11] and in one series has been shown to provide excellent results in selected patients with one to 10 brain lesions without prophylactic whole-brain radiotherapy [12]. Even in our case report, treatment with gamma knife proved to be successful in providing good local disease control.

This case report represents the fifth reported case of MCC metastasis to the spinal canal (Table 2) [13-17]. However, our patient had both epidural and intradural tumors. To the best of our knowledge, this is the first case report in the literature on intradural extension of MCC. The detection of an intradural tumor disqualified our patient for decompressive laminectomy, which has previously been proven to be an effective treatment for spinal and epidural metastasis compared with radiotherapy or chemotherapy alone [16]. Unlike the other reports of the intraspinal epidural metastases [13,14], our patient did not show evidence of osseous vertebral metastases, which is a usual source of the epidural tumor extension. The possible explanation for the route of the intradural metastases is the meningeal spread of the large meningeal supratentorial lesion, which gave rise to the cerebellar leptomeningeal metastases detected shortly before the detection of intradural metastasis. Consistent with other reports of poor prognosis of intraspinal metastases [12,13,16], our patient showed evidence of progressive systemic metastatic spread despite initial improvement with reduction of tumor size intracranially and regression of the retroperitoneal lymph nodes.

**Table 2:** Intraspinal metastases of Merkel cell carcinoma reported in the literature

	Year	Age/gender	Other metastasis	Intraspinal involvement	Level	Time since detection of the primary tumor	Survival after diagnosis
Moayed et al [13]	2000	70/M	LN	Epidural*	S	9 months	--
Turgut et al [14]	2002	63/M	LN	Epidural*	L5-S1	0	2 months
Turgut et al [15]	2004	65/M	--	Epidural	L5-S1	--	--
Vijay et al [16]	2007	57/F	LN	Epidural	T8, L4, S1	0	1 month
Ng et al [17]	2010	73/M	-	Epidural	T6	6 months	7 months
Present case	2010	65/M	LN	Epidural, intradural	T11-S	8.5 months^†^	8 months

* Associated osseous involvement.

^† ^In the present case, the given time interval represents the time between the first presentation and the detection of the intraspinal metastases as the primary site of the tumor; in this case, the time remain unknown.

F, female; LN, lymph node; M, male.

## Conclusion

Our patient with extensive CNS, abdominal and inguinal metastases showed a good initial response to radiation therapy. However, evidence of progressive metastatic spread was demonstrated already six months after the onset of the patient's symptoms. This is the first report in the literature of spinal intradural metastases of MCC, which further contributed to worsening the patient's prognosis because it restrained a debulking and decompressive laminectomy.

## Consent

Written informed consent was obtained from the patient's next of kin for publication of this case report and the accompanying images. A copy of the written consent is available for review by the Editor-in-Chief.

## Competing interests

The authors declare that they have no competing interests.

## Authors' contributions

KAK conceived the idea of the study. All authors participated in data collection. KAK wrote the manuscript. All authors read and contributed to the editing of the manuscript and gave their approval of the final manuscript.
